# A Multifunctional PEEK Composite Scaffold with Immunomodulatory, Angiogenic, and Osteogenic Properties for Enhanced Bone Regeneration

**DOI:** 10.3390/polym17091206

**Published:** 2025-04-28

**Authors:** Mengen Zhao, Han Yang, Qianwen Yang, Chao Zhang, Jie Liu, Zhaoying Wu, Lijun Wang, Wei Zhang, Bing Wang, Wenliang Liu

**Affiliations:** 1 Shenzhen Institute for Drug Control, Shenzhen Testing Center of Medical Devices, Shenzhen 518057, China; zhaomen3@mail.sysu.edu.cn (M.Z.); yanghan@szidc.org.cn (H.Y.); wanglijun8706@hotmail.com (L.W.); zhangwei@szidc.org.cn (W.Z.); 2 School of Biomedical Engineering, Shenzhen Campus, Sun Yat-sen University, Shenzhen 518107, China; yangxw29@mail2.sysu.edu.cn (Q.Y.); zhchao9@mail.sysu.edu.cn (C.Z.); liujie56@mail.sysu.edu.cn (J.L.); wuzhy37@mail.sysu.edu.cn (Z.W.)

**Keywords:** polyetheretherketone, immunomodulation, angiogenesis, osteogenesis, bone regeneration

## Abstract

Polyetheretherketone (PEEK) is a widely used material in bone tissue engineering due to its favorable mechanical properties and radiolucency. However, its bioinert nature and lack of osteogenic activity restrict its ability to support effective bone regeneration. In this study, a novel APS-coated plasma-treated sulfonated bioactive PEEK scaffold (APS/PSBPK) was developed to overcome these limitations. The scaffold integrates strontium-doped bioactive glass (SrBG) to enhance biocompatibility and osteogenic potential, while astragalus polysaccharide (APS) was incorporated via plasma cleaning to modulate immune responses and promote vascularization. In vitro studies demonstrated that the APS/PSBPK scaffold facilitates M2 macrophage polarization, reduces pro-inflammatory cytokines, and enhances the secretion of anti-inflammatory factors. It also promotes endothelial cell migration and angiogenesis while supporting the adhesion, proliferation, and osteogenic differentiation of rBMSCs. In vivo experiments revealed that the scaffold effectively regulates the immune microenvironment, promotes vascularization, and accelerates bone regeneration. Thus, the APS/PSBPK composite scaffold serves as a multifunctional biomaterial with significant potential for applications in bone repair and regeneration by combining immunomodulation, angiogenesis, and osteogenesis.

## 1. Introduction

Traditionally, artificial bone repair materials have been designed to replicate the structural and mechanical properties of natural bone tissue, such as stiffness, porosity, and mechanical strength, to provide support for damaged areas [1,2]. While these static characteristics are essential, bone repair is a highly dynamic process that involves complex interactions among immune responses, vascularization, and osteogenesis [3,4,5]. In particular, the immune system plays a pivotal role in regulating inflammation and facilitating tissue regeneration, highlighting the need for biomaterials that actively modulate the bone healing microenvironment [6,7,8,9,10]. Despite this, many current bone repair materials do not fully address the dynamic nature of the bone healing process, focusing instead on static tissue replacement. This results in poor integration between the synthetic material and the native tissue, with discrepancies between in vitro and in vivo performance, ultimately leading to suboptimal outcomes in clinical applications.

Thus, a key limitation in the design of conventional bone repair materials lies in their inability to simulate the dynamic interactions that occur during bone regeneration [4,11,12]. Without the ability to interact with and modulate the biological environment, the materials are often encapsulated by fibrous tissue rather than being integrated into the bone, leading to implant loosening or failure over time [13,14,15]. Therefore, there is growing recognition of the need for new design strategies that incorporate dynamic elements into bone repair materials. By mimicking the dynamic processes of bone healing, such as immune modulation and angiogenesis, new materials can potentially improve integration and promote faster and more effective bone regeneration.

In response to these challenges, a combination of advanced materials has gained attention for their potential to overcome the limitations of static bone repair materials. Polyetheretherketone (PEEK) has been widely used in orthopedic applications due to its favorable mechanical properties and excellent biocompatibility. PEEK has a modulus of elasticity similar to that of cortical bone, making it well suited for load-bearing applications [16,17,18]. However, PEEK is biologically inert, meaning it does not actively promote bone regeneration or integration with surrounding tissues. This limitation has driven interest in strategies to enhance its bioactivity, such as surface modification or incorporation into composite materials with bioactive components [19,20,21,22,23].

Bioactive glass has demonstrated significant potential in bone tissue engineering, forming a direct chemical bond with surrounding bone tissue, thereby promoting osteogenesis and enhancing implant integration [24]. Bioactive glass is composed of a silica-based network that dissolves in physiological conditions, releasing ions that stimulate cellular responses critical to bone healing [25]. In particular, ion-doped bioactive glass, such as those containing strontium (Sr^2+^), has been shown to further enhance osteogenic activity and support the regeneration of bone tissue [26,27]. The incorporation of bioactive glass into composite scaffolds holds the potential to create a material that combines the mechanical strength of PEEK with the bioactivity of bioactive glass, addressing both structural and biological aspects of bone repair. Zheng et al. [28] reported that zinc-doped bioactive glass-functionalized polyetheretherketone enhances the biological response in bone regeneration. Similarly, Liu et al. [29] demonstrated that magnesium bioactive glass hybrid-functionalized polyetheretherketone exhibits immunomodulatory properties, guiding cell fate and promoting bone regeneration.

In addition to mechanical strength and bioactivity, immunomodulation is a key factor in the design of next-generation bone repair materials. *Astragalus polysaccharide* (APS), a bioactive compound derived from the traditional Chinese medicinal herb Astragalus, has garnered attention for its immunomodulatory properties [30,31]. APS has been shown to regulate macrophage polarization, a critical step in the inflammatory phase of bone healing. By shifting macrophages from a pro-inflammatory (M1) to an anti-inflammatory (M2) phenotype, APS can reduce excessive inflammation and promote tissue regeneration [32,33,34]. Furthermore, APS has been demonstrated to support angiogenesis by enhancing the proliferation and migration of endothelial cells, which is essential for the formation of new blood vessels during the healing process [35]. The combination of APS with PEEK and bioactive glass presents a novel approach to developing a bone repair scaffold that not only supports the mechanical and structural requirements of bone healing but also actively modulates the immune response and promotes vascularization.

The objective of this study is to develop an innovative bioactive, three-dimensional porous composite scaffold (APS/PSBPK) that mimics the dynamic bone repair process, enabling the controlled release of anti-inflammatory drugs and metal ions to regulate the bone microenvironment. The scaffold utilizes PEEK as a base to provide early mechanical support and incorporates Sr-doped bioactive glass to improve bioactivity, promote osseointegration. Plasma treatment enables the surface loading of astragalus polysaccharides (APS), which modulate the early immune response and promote vascularization, optimizing the implant microenvironment for improved bone tissue repair. In vitro cell assays were conducted to evaluate the immunomodulatory, angiogenic, and osteogenic properties of the APS/PSBPK composite scaffold. In vivo subcutaneous implantation experiments were performed to assess the immunomodulatory and angiogenic effects of the composite scaffold. Furthermore, the osteogenic performance of the APS/PSBPK scaffold was investigated using a rat femoral defect model.

## 2. Materials and Methods

### 2.1. Fabrication of Composite Scaffolds

In this study, SrBG nanoparticles were synthesized following a sol–gel method based on our previous research [36]. In summary, a silica sol was prepared using a volume ratio of TEOS:EtOH:H_2_O:NH_3_·H_2_O = 1:12:5:1.5. Calcium and strontium precursors, Ca(NO_3_)_2_·4H_2_O and Sr(NO_3_)_2_, were added to achieve a final composition of 88% SiO_2_, 6% CaO, and 6% SrO. The mixture was stirred for 90 min, after which the precipitate was collected and dried at 100 °C for 6 h. This was followed by calcination at 700 °C for 2 h, yielding SrBG nanoparticles.

A porous PEEK scaffold (dimensions: Φ12 × 2 mm for in vitro experiments, Φ3 × 8 mm for in vivo experiments) was produced using a melt deposition technique with 1.75 mm diameter PEEK wire. The printing parameters included a nozzle temperature of 450 °C, hotbed temperature of 150 °C, cavity temperature of 90 °C, fiber thickness of 400 µm, pore size of 400 µm, and a printing speed of 20 mm/s. After printing, the scaffold was sequentially cleaned with acetone, ethanol, and distilled water, followed by air drying at room temperature. To create the PEEK/SrBG (BPK) composite scaffold, 3% SrBG nanoparticles were blended into the PEEK matrix.

The scaffolds were then immersed in concentrated sulfuric acid and stirred at 500 rpm for 6 min to obtain sulfonated BPK (SBPK) scaffolds. These were subjected to hydrothermal treatment at 100 °C for 4 h, followed by plasma treatment using a Sunjune VP-Q5 plasma machine (Sunjune, Guangzhou, China) for 100 s, resulting in plasma-treated SBPK (PSBPK) scaffolds. Finally, the PSBPK scaffolds were immersed in an APS solution (5 mg/mL) and stirred at room temperature for 6 h, producing APS-coated PSBPK (APS/PSBPK) scaffolds.

### 2.2. Characterization of Scaffolds

The morphology of the scaffolds and elemental distribution on the scaffold surface were examined using a TESCAN MIRA LMS field-emission scanning electron microscope (FESEM, TESCAN, Brno, Czech Republic). The composition of the scaffolds was assessed using Fourier transform infrared spectroscopy (FTIR, Nicolet iS5, Thermo Fisher Scientific, Waltham, MA, USA) with a spectral resolution of 2 cm^−1^ over the 400–4000 cm^−1^ wavelength range, and X-ray diffraction (XRD, Rigaku Ultima IV, Rigaku Corporation, Tokyo, Japan) was performed using Cu Kα radiation at 40 kV and 40 mA. X-ray photoelectron spectroscopy (XPS, ESCALAB 250XI, Thermo Fisher Scientific, Waltham, MA, USA) was utilized to analyze surface elemental changes in the scaffolds, employing an Al Kα X-ray source with an energy step size of 1.000 eV.

The surface laser intensity of the samples was measured using a VK-150K laser microscope (KEYENCE, Osaka, Japan) operating at a wavelength of 658 nm. The captured laser intensity data were processed using VK-H1XAC software (Version 2.1.3.89) to reconstruct three-dimensional images and map the surface height distribution.

### 2.3. Cell Morphology and Proliferation

Cell proliferation on the scaffolds was evaluated using Cell Counting Kit-8 (CCK-8, Beyotime, Shanghai, China). In brief, 2.0 × 10^4^ rBMSCs were seeded onto the scaffolds and incubated for 1, 3, and 5 days. After each incubation period, the cell culture medium was replaced with 1.0 mL of fresh medium containing 100 μL of CCK-8 solution per well. The cells were then incubated for an additional 2 h, and absorbance was measured at 450 nm using a Gene5 Microplate Reader (BioTek Synergy, Winooski, VT, USA) to determine cell viability. Additionally, live/dead staining was performed on the cells seeded onto the scaffolds, and confocal laser scanning microscopy (CLSM, NIS-Elements, Nikon, Tokyo, Japan) was used to observe and capture images at 1 and 3 days.

To assess cell morphology, rBMSCs were seeded onto the scaffold surfaces and incubated for 24 h. The cells were then fixed with 4% paraformaldehyde for 30 min after washing three times with PBS. Immunofluorescent staining was performed using TRITC-conjugated Phalloidin to stain the cytoskeleton and DAPI to stain the nuclei. Cell morphology was visualized and captured using confocal laser scanning microscopy.

### 2.4. Alkaline Phosphatase (ALP) and Alizarin Red S Staining of rBMSCs

A total of 4 × 10^4^ rBMSCs were seeded into a 12-well plate, with the composite scaffold positioned in the transwell inserts. After incubating at 37 °C for 24 h to allow for cell attachment, the regular culture medium was replaced with osteogenic induction medium, Following 7, 10, and 14 days of induction, the wells were rinsed three times with PBS. For ALP quantification, 400 μL of 0.2% Triton X-100 was added to each well for 30 min to lyse the cells, and the resulting solution was collected in 1.5 mL centrifuge tubes. The samples were centrifuged at 7000 rpm for 2 min, and the supernatant was used to measure ALP activity and total protein content, with ALP activity calculated according to standard protocols.

ALP staining was further visualized using a BCIP/NBT staining kit on days 7, 10, and 14 to detect early osteogenic markers. On day 14 and 21, calcium deposition, a later marker of osteogenesis, was assessed using Alizarin Red S staining. The cells were fixed with 4% paraformaldehyde for 30 min, followed by immersion in the staining solutions for 30 min. Excess stains were removed by washing with distilled water, and the staining patterns were analyzed under a light microscope. After imaging, the calcium-binding dye was extracted with 200 μL of 0.1 M cetylpyridinium chloride, and the absorbance of the extracted solution was measured at 562 nm to quantify calcium content.

### 2.5. Flow Cytometry

RAW 264.7 cells (2.0 × 10^5^) were seeded onto the scaffold and cultured for 1 and 3 days. Then, Cells were digested with 200 μL of trypsin for 5 min, collected, and centrifuged at 1000 rpm for 5 min. After discarding the supernatant, the cells were resuspended in 300 μL PBS, centrifuged again, and incubated with 300 μL PE-anti-CD86 antibody (1:400 in PBS) at 4 °C for 20 min. The cells were centrifuged, the supernatant discarded, then incubated with 300 μL FITC-anti-CD206 antibody (1:400 in 0.1% saponin) for 20 min. After another wash, the stained cells were analyzed by flow cytometry to detect the expression of M1 (CD86) and M2 (CD206) markers.

### 2.6. In Vitro Angiogenic Activity

To evaluate human umbilical vein endothelial cell (HUVEC) migration, a wound healing assay was conducted using the following method: First, 5 × 10^5^ HUVECs were seeded in a 12-well plate and co-cultured with scaffolds for 12 h. After the incubation period, a scratch wound was created using a sterile 200 μL pipette tip. The culture medium was then replaced with serum-free medium, and the cells continued to co-culture with the scaffolds. To visualize the cells, a live/dead staining kit was used according to the manufacturer’s instructions. After staining, the cells were observed under a Leica optical microscope at specified time intervals to monitor the closure of the scratch wound. Images were captured to evaluate the migration of HUVECs into the scratched area, providing insights into their migratory response to the scaffolds.

To assess the vascularization potential of the scaffolds, Matrigel angiogenesis assays were performed. First, 96-well plates were precoated with 20 μL of chilled Matrigel per well and allowed to set for 30 min. HUVECs cultured with different scaffolds for 3 days were then digested and seeded at a density of 8000 cells per well. After incubating for 3 and 6 h, the cells were stained using a live/dead kit for fluorescent imaging. The Matrigel cultures were observed under a microscope, and the parameters of tube formation were quantified using ImageJ software (Version 1.51j8) to obtain average values.

### 2.7. In Vivo Experiment

#### 2.7.1. Subcutaneous Implantation Model

SD rats were used for subcutaneous implantation of sterilized scaffolds (PEEK, SBPK, APS/PSBPK, Ø3 × 8 mm). After anesthesia with 10% sodium pentobarbital (50 mg/kg), the rats’ backs were shaved and disinfected. Small incisions were made, and the scaffolds were implanted subcutaneously on both sides, followed by wound suturing and disinfection. At 14 and 28 days, tissues around the implants were collected, fixed, paraffin-embedded, and analyzed using hematoxylin & eosin (H&E) and immunofluorescence staining.

#### 2.7.2. Rat Skull Defect Models

SD rats were used to establish cranial defect models. Sterilized implants (PEEK, SBPK, APS/PSBPK, Ø3 × 8 mm) were inserted into the cranial defects, and samples were collected at 6 and 12 weeks post-surgery. For imaging, the fixed samples were scanned using a Skyscan 1176 µ-CT scanner at 55 kV and 145 µA, with a resolution of 12 µm per sample. Following 3D reconstruction, bone mineral density (BMD), bone volume fraction (BV/TV), trabecular number (Tb.N), and trabecular separation (Tb.Sp) in the defect regions were measured. New bone formation was quantified using CT software (Version 1.5.6.2). Histological analysis was conducted on paraffin-embedded samples fixed in 4% paraformaldehyde. H&E and Masson’s trichrome staining were performed to evaluate tissue integration and bone regeneration.

## 3. Results and Discussion

### 3.1. The Characterization of Scaffolds

**Figure 1:**
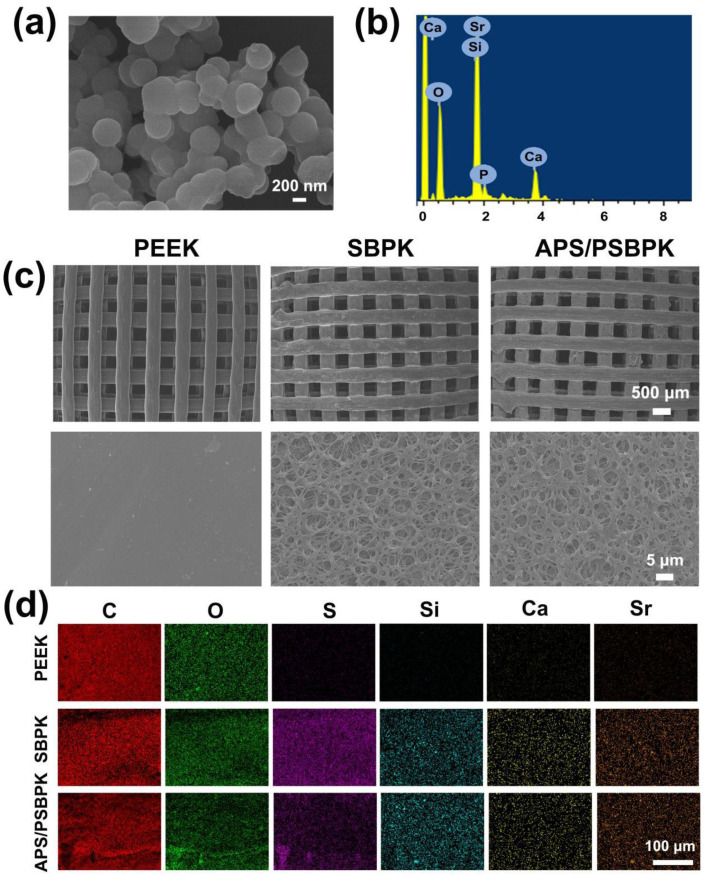
Surface morphology (**a**) and EDS spectra (**b**) of SrBG nanoparticles. Surface morphology (**c**) and EDS spectra (**d**) of PEEK, SBPK, and APS/PSBPK scaffolds.

The surface morphology and elemental composition of SrBG, PEEK, SBPK, and APS/PSBPK scaffolds were detected by FESEM and EDS analysis. The SrBG nanoparticles exhibit a spherical morphology with uniform particle sizes of approximately 200 nm (Figure 1a). The corresponding EDS spectrum (Figure 1b) clearly reveals distinct peaks for Si, Ca, O, and Sr, confirming the composition of the SrBG nanoparticles.

FESEM images (Figure 1c) demonstrate the structural differences among the scaffolds. The PEEK scaffold exhibits a well-defined, regular square channel structure (~400 × 400 µm) with smooth surfaces. In contrast, the SBPK scaffold shows a roughened three-dimensional porous structure induced by sulfuric acid sulfonation, which facilitates improved cell adhesion, migration, and nutrient transport. Following the loading of APS, the APS/PSBPK scaffold successfully maintains the interconnected three-dimensional porous architecture, ensuring structural stability and bioactivity.

The EDS mapping images (Figure 1d) reveal the elemental distribution on the scaffold surfaces. C and O are uniformly distributed across all scaffolds, consistent with the composition of PEEK. The presence of S in SBPK, P and APS/PSBPK scaffolds confirms the successful sulfonation treatment applied to expose SrBG particles on the scaffold surface. Furthermore, Si, Ca, and Sr are observed in SBPK and APS/PSBPK, indicating the incorporation of SrBG within the scaffold structure. This distribution of bioactive elements suggests that APS/PSBPK scaffolds possess enhanced potential for bone regeneration by supporting mineralization and modulating the local bone microenvironment.

**Figure 2:**
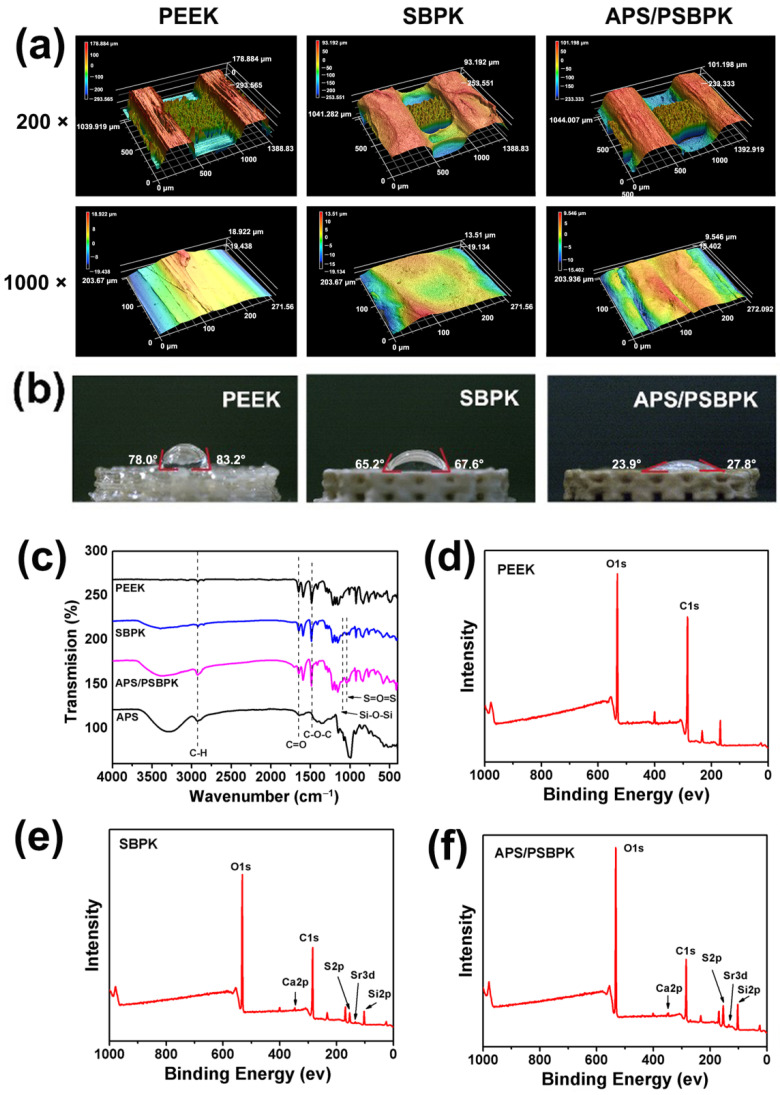
Laser microscope 3D images (**a**), water contact angles (**b**), FTIR spectra (**c**), and XPS spectra (**d**–**f**) of PEEK, SBPK, and APS/PSBPK scaffolds.

The surface topography of various scaffolds was examined using three-dimensional (3D) imaging at magnifications of 200× and 1000× of PEEK, SBPK, and APS/PSBPK scaffolds (Figure 2a). The roughness average (Ra) values indicated a significant variation in surface roughness, which is critical for cellular interactions. PEEK exhibited the smoothest surface with an Ra of 0.028 ± 0.006 μm, while SBPK presented significantly higher Ra values of 0.573 ± 0.008 μm. The APS/PSBPK scaffold shows an intermediate Ra of 0.230 ± 0.014 μm, suggesting that varying compositions and processing methods effectively alter the surface characteristics.

The wettability of the scaffolds was assessed by measuring the contact angles of water droplets on their surfaces (Figure 2b). PEEK shows a contact angle of 80.47 ± 2.4°, indicating moderate hydrophobicity. SBPK (with a contact angle of 60.43 ± 2.5°), is slightly more hydrophilic. In contrast, APS/PSBPK exhibited significantly lower contact angles (25.20 ± 3.0°), highlighting their high hydrophilicity. Higher hydrophilicity, as seen in APS/PSBPK, is beneficial for cell adhesion, nutrient absorption, and protein adsorption, which are vital for enhancing cellular activities during bone repair. This property may accelerate the healing process by fostering a more favorable microenvironment for osteoconductivity.

The FTIR spectra of the scaffolds (Figure 2c) revealed that the PEEK scaffold shows characteristic peaks corresponding to C–H (aromatic) stretching vibrations and C=O stretching at specific wavenumbers, consistent with its polymeric structure. For the SBPK scaffold, additional peaks appear in the range of 1200–1000 cm^−1^, corresponding to S=O symmetric and asymmetric stretching vibrations, confirming the successful introduction of sulfonic acid groups during the sulfuric acid sulfonation process. These functional groups play a crucial role in improving hydrophilicity and facilitating the exposure of SrBG particles for enhanced bioactivity. In the APS/PSBPK scaffold, new peaks associated with Si–O–Si bonds (at ~1050 cm^−1^) are observed, which can be attributed to the presence of SrBG particles loaded onto the scaffold surface. The combination of sulfonation and SrBG particle deposition creates a surface with multiple bioactive functional groups, enhancing its osteoconductive and osteoinductive properties.

The XPS spectra of the scaffolds (Figure 2d–f) demonstrated that the PEEK scaffold exhibits only the presence of C 1s and O 1s peaks, consistent with its polymeric composition [21]. In addition to C 1s and O 1s peaks, new peaks corresponding to S2p (sulfur), Si2p (silicon), Sr3d (strontium), and Ca2p (calcium) are observed in SBPK and APS/PSBPK scaffolds [37,38,39,40]. The presence of sulfur confirms successful sulfonation, while the detection of Si, Sr, and Ca validates the exposure of SrBG particles on the scaffold surface. These bioactive elements are essential for promoting mineralization and stimulating osteogenic activity.

The compressive strength and modulus of PEEK, SBPK, and APS/PSBPK scaffolds were evaluated and are presented in Table S1. The compressive strengths of PEEK, SBPK, and APS/PSBPK scaffolds were 35.24 ± 0.65, 32.10 ± 1.12, and 35.56 ± 0.04 MPa, respectively, while the corresponding moduli were 585.33 ± 8.14 MPa, 527.67 ± 7.02 MPa, and 531.00 ± 3.60 MPa. The mechanical properties of the composite PEEK scaffolds fall within the range of human trabecular bone (0.3~3.2 GPa), suggesting good biomechanical compatibility and potential for application in bone tissue engineering and non-load-bearing bone defect repair.

### 3.2. In Vitro Mineralization and Ion/Drug Release

To investigate the mineralization ability of the PEEK, SBPK, and APS/PSBPK scaffolds in vitro, these scaffolds were immersed in simulated body fluid (SBF) for 14 days. The SEM images (Figure S1a) demonstrate significant differences in surface morphology after immersion. The PEEK scaffold remained largely unchanged, displaying a smooth surface with minimal alterations. In contrast, the surfaces of SBPK and APS/PSBPK scaffolds were covered with spherical deposits, respectively, indicative of mineral deposition. Furthermore, EDS analysis (Figure S1b) revealed that the PEEK scaffold surface was composed of only carbon (C) and oxygen (O), signifying little or no mineral deposition. In contrast, the SBPK and APS-PSBPK scaffolds showed prominent peaks of calcium (Ca) and phosphorus (P), indicating the formation of apatite-like minerals on their surfaces, which is critical for promoting osteogenesis and bone regeneration in bone tissue engineering applications.

**Figure 3:**
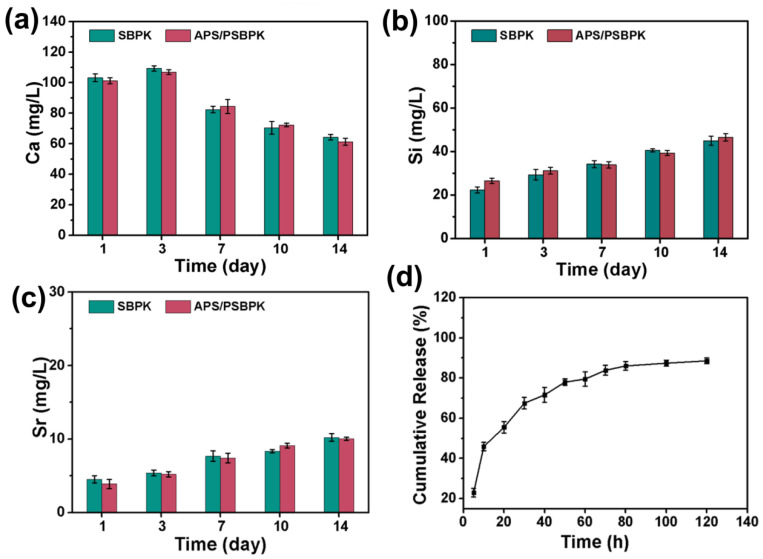
(**a**–**c**) The ion release of SBPK and APS/PSBPK scaffolds in SBF solution. (**d**) Cumulative drug release behavior of APS/PSBPK scaffold in PBS solution.

To evaluate the ion release behavior of the SBPK and APS/PSBPK scaffolds in a simulated body fluid (SBF) solution, the concentrations of Ca^2+^, SiO_3_^2−^, and Sr^2+^ ions were measured over a 14-day period (Figure 3a–c). The concentration of Ca^2+^ increased within the first three days, likely due to the release of calcium ions from the scaffold. However, after three days, the concentration began to decrease, which is attributed to the formation of apatite on the scaffold surface. In contrast, the concentrations of SiO_3_^2−^ (Figure 3b) and Sr^2+^ (Figure 3c) continued to rise over time, indicating the ongoing degradation of the SrBG component, resulting in the sustained release of silicon and strontium ions, both crucial for promoting bone regeneration.

In addition, the cumulative release profile of APS from the APS/PSBPK scaffolds in PBS solution (Figure 3d) demonstrated a sustained release over time, reaching approximately 90% by 120 h. This slow and controlled release of APS indicates the scaffold’s capability to modulate immune responses, promote angiogenesis, and support osteogenic differentiation, which are critical factors in facilitating effective bone repair. This design highlights the potential of APS/PSBPK scaffolds for enhancing bone regeneration through immunomodulation and osteoinductive properties.

### 3.3. Effect of Scaffolds on Cell Proliferation and Osteogenic Differentiation of rBMSCs

**Figure 4:**
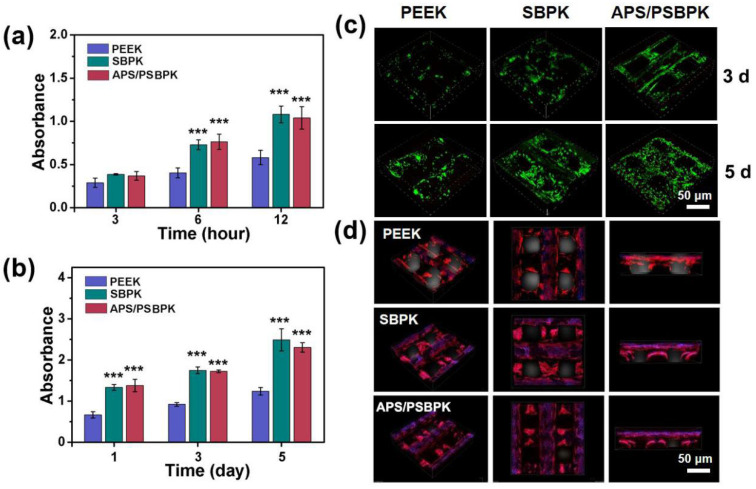
Cell adhesion (**a**) and proliferation (**b**) of rBMSCs cultured on the surface of PEEK, SBPK, and APS/PSBPK scaffolds at different time points. Live/dead staining (**c**) of rBMSCs cultured on the surface of PEEK, SBPK, and APS/PSBPK scaffolds after 3 and 5 days. (Green:live rBMSCs; Red: dead rBMSCs). CLSM images (**d**) of rBMSCs cultured on the surface of PEEK, SBPK, and APS/PSBPK scaffolds for 5 days (Blue: nuclei; Red: F-actin). *** *p* < 0.001.

The CCK-8 assay was employed to evaluate the effects of PEEK, SBPK, and APS/PSBPK scaffolds on cell adhesion and proliferation. The results (Figure 4a) indicated that there were no significant differences in cell adhesion among all scaffolds at 3 h. However, at 6 and 12 h, the absorbance values for rBMSCs on the SPBK and APS/PSPBK scaffolds were significantly higher compared to the PEEK scaffold, with the APS/PSPBK group showing the highest absorbance. This suggests that the APS/PSPBK scaffold is more favorable for cell adhesion. As time progressed, the proliferation rate of rBMSCs gradually increased on all scaffolds (Figure 4b). Notably, the proliferation rates on the SPBK and APS/PSPBK scaffolds were significantly higher than that on the PEEK scaffold (*p* < 0.001), with the APS/PSPBK exhibiting the highest rate. These findings imply that the SPBK and APS/PSPBK scaffolds enhanced the proliferation of rBMSCs more effectively than the PEEK, with APS/PSPBK scaffold showing the most pronounced effect.

Live/dead staining images (Figure 4c) reveal the viability of cells cultured on PEEK, SBPK, and APS/PSBPK scaffolds over 3 and 5 days. Green fluorescence, indicating live cells, shows a noticeable increase in cell coverage over time, particularly on the APS/PSBPK scaffold. After 3 days, all scaffolds supported cell adhesion, but by day 5, the APS/PSBPK scaffold exhibited the highest density of live cells, suggesting superior cell proliferation and survival. The distribution of live cells on APS/PSBPK was more uniform compared to the PEEK and SBPK scaffolds, highlighting the bioactivity of the scaffold, likely due to the release of osteoinductive ions that support cell growth.

Confocal laser scanning microscopy (CLSM) images illustrated the adhesion and spreading of rBMSCs on the various scaffold surfaces after 5 days of culture (Figure 4d). Compared to the PEEK scaffold, both SPBK and APS/PSBPK scaffolds displayed improved cell morphology. Among them, the APS/PSPBK scaffold had the highest number of adhered cells, the best cell spreading, and the largest overall volume. Therefore, the APS/PSPBK demonstrated a greater capacity to induce cell growth and promote cell adhesion and proliferation.

**Figure 5:**
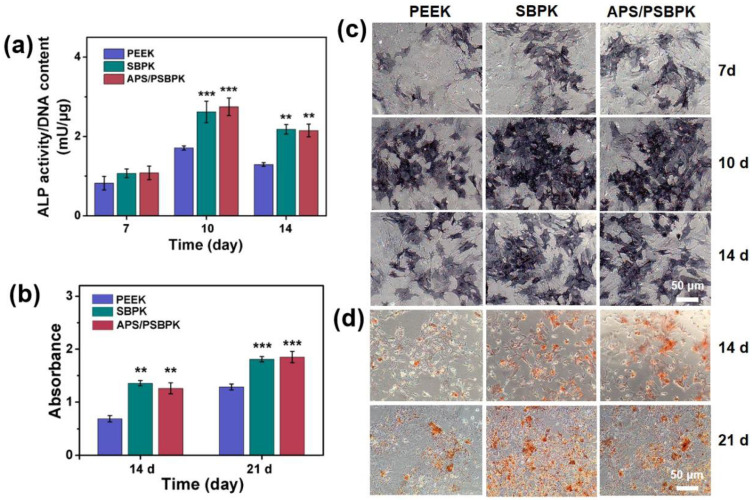
ALP activity (**a**) and ALP staining (**c**) of rBMSCs cultured with PEEK, SBPK, and APS/PSBPK scaffolds 7, 10, and 14 days; Alizarin Red S staining (**d**) and quantitative analysis (**b**) of rBMSCs cultured with PEEK, SBPK, and APS/PSBPK scaffolds at 14 and 21 days. (** *p* < 0.01, *** *p* < 0.001).

The ALP activity and staining results (Figure 5a,c) revealed a significant increase in ALP expression in the SBPK and APS/PSBPK group, highlighting its superior osteogenic potential compared to the PEEK group (*p* < 0.01). In terms of mineralization, Alizarin Red S staining and quantitative analysis (Figure 5b,d) indicated a more pronounced formation of mineralization nodules in the SBPK and APS/PSBPK group compared to the PEEK and SBPK groups. The inhibition of mineralized nodule formation was particularly evident in the PEEK group, whereas SBPK showed a more moderate effect. However, the APS/PSBPK group notably enhanced the formation of mineralized nodules, signifying its effectiveness in fostering osteogenic activity. Quantitative assessments further reinforced these observations, showing that the APS/PSBPK group had a significantly higher count of mineralized nodules at both day 14 and day 21, compared to the other groups (*p* < 0.01). This suggests that while SBPK and APS/PSBPK share certain similarities, the ability of APS/PSBPK to promote bone differentiation is markedly superior to that of PEEK.

In conclusion, our findings strongly indicate that the SBPK and APS/PSBPK group not only enhances ALP activity but also promotes extensive mineralization, thus demonstrating its potential as a more effective substrate for bone tissue engineering applications compared to PEEK and SBPK. This positions APS/PSBPK as a promising candidate for future studies aimed at optimizing bone regeneration strategies.

### 3.4. Effect of the Scaffolds on Macrophage Polarization and Cytokine Secretion

**Figure 6:**
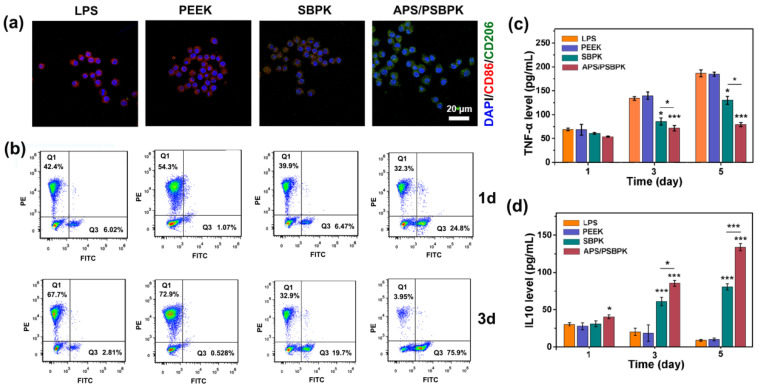
(**a**) Immunofluorescent staining for macrophage polarization markers (CD86 and CD206) on LPS, PEEK, SBPK, and APS/PSBPK scaffolds after 1 and 3 days of culture; (**b**) flow cytometry analysis of CD86 and CD206 expression on macrophages cultured on different scaffolds; (**c**) TNF-α levels and (**d**) IL-10 levels in macrophages on LPS, PEEK, SBPK, and APS/PSBPK scaffolds at 1, 3, and 5 days, as measured by ELISA. (* *p* < 0.05, *** *p* < 0.001).

To explore the immunomodulatory effects of PEEK, SBPK, and APS/PSBPK scaffolds on RAW264.7 macrophages, immunofluorescence staining (Figure 6a) was conducted to visualize the expression of M1 macrophage marker CD86 (red) and M2 macrophage marker CD206 (green) after 3 days of culture on the different scaffolds. Macrophages cultured on the PEEK scaffold exhibited high expression of CD86, indicating a predominant M1 polarization. This suggests that PEEK scaffolds tend to promote a pro-inflammatory response, as M1 macrophages are associated with the secretion of inflammatory cytokines that may hinder tissue regeneration. A moderate reduction in CD86 expression was observed on the SBPK scaffold, accompanied by an increase in CD206 expression, suggesting a partial shift towards M2 polarization. The ability of the SBPK scaffold to induce M2 macrophage polarization highlights its potential to attenuate the inflammatory response and support tissue repair. The APS/PSBPK scaffold demonstrated the most pronounced promotion of M2 polarization, with macrophages exhibiting high expression of CD206 and minimal expression of CD86. This indicates that the bioactive ion and APS release from APS/PSBPK scaffolds create a favorable environment for anti-inflammatory macrophage polarization, which is crucial for tissue healing and integration.

Flow cytometry (Figure 6b) was used to quantify the proportion of M1 (CD86-positive) and M2 (CD206-positive) macrophages cultured on the different scaffolds at 1 and 3 days post-seeding. Macrophages on the PEEK scaffold showed the highest percentage of CD86-positive (M1) cells (54.3%), confirming its tendency to promote a pro-inflammatory phenotype. In contrast, the APS/PSBPK scaffold significantly increased the proportion of CD206-positive (M2) cells, with 24.8% of the cells being M2-polarized. This suggests that APS/PSBPK scaffolds begin to promote M2 polarization as early as 1 day post-seeding. By day 3, macrophages on the PEEK scaffold still exhibited a high percentage of M1 cells (72.9%), while the APS/PSBPK scaffold continued to promote a strong M2 response, with over 75.9% of the cells being CD206-positive. This result highlights the sustained ability of the APS/PSBPK scaffold to drive M2 macrophage polarization over time, whereas the PEEK scaffold continued to favor M1 polarization. The SBPK scaffold displayed intermediate effects, reducing the percentage of M1 macrophages while modestly increasing the proportion of M2 macrophages compared to PEEK, but to a lesser extent than APS/PSBPK.

To further explore the immunomodulatory effects of the scaffolds, the levels of tumor necrosis factor-alpha (TNF-α), a pro-inflammatory cytokine, and interleukin-10 (IL-10), an anti-inflammatory cytokine, were measured in the culture supernatant at 1, 3, and 5 days post-seeding using ELISA (Figure 6c). After 1 day of culture, no significant differences in TNF-α levels were observed across the groups. However, by days 3 and 5, the APS/PSBPK scaffold significantly suppressed TNF-α secretion compared to the PEEK and SBPK scaffolds (*p* < 0.001). TNF-α levels in macrophages cultured on APS/PSBPK were reduced by more than 50% by day 5, indicating a potent inhibition of the pro-inflammatory response. This reduction suggests that APS/PSBPK scaffolds can effectively minimize the inflammatory response, which is essential for promoting tissue healing and minimizing fibrous tissue encapsulation. IL-10 levels remained relatively low after 1 day of culture across all scaffolds. By day 3, macrophages on the SBPK and APS/PSBPK scaffolds exhibited a significant increase in IL-10 secretion compared to the PEEK scaffold, with APS/PSBPK showing the highest IL-10 levels. By day 5, the APS/PSBPK scaffold had nearly doubled IL-10 secretion compared to SBPK and PEEK scaffolds (*p* < 0.001). IL-10 is a key anti-inflammatory cytokine that promotes tissue repair by downregulating pro-inflammatory responses and enhancing the resolution of inflammation. The robust IL-10 secretion induced by APS/PSBPK scaffolds suggests their superior capacity to create a pro-healing immune environment.

In conclusion, APS/PSBPK scaffolds demonstrate superior immunomodulatory effects by promoting M2 macrophage polarization and enhancing IL-10 secretion, while suppressing pro-inflammatory TNF-α production. These properties make APS/PSBPK a promising candidate for bone tissue engineering applications, where controlling the immune response is critical for successful tissue regeneration and implant integration. Further studies on in vivo models would be necessary to confirm these results and explore the long-term performance of these scaffolds in clinical applications.

In the field of bone regeneration, the interaction between implant materials and the host immune system is crucial for determining the success of implant integration. Specifically, the modulation of macrophage polarization plays a pivotal role in this process [41]. Bioactive ions, such as Sr^2+^, have demonstrated substantial effects on both immunomodulation and bone healing. Sr^2+^, in particular, has been well-studied for its ability to promote bone formation while simultaneously modulating immune responses. Several studies have shown that Sr^2+^ can suppress the pro-inflammatory activity of M1 macrophages and promote the differentiation of M2 macrophages. By doing so, Sr^2+^ helps to reduce the levels of inflammatory cytokines like TNF-α and IL-6, which are often associated with poor bone healing due to prolonged inflammation. Additionally, Sr^2+^ increases the secretion of anti-inflammatory cytokines such as IL-10, which fosters an environment conducive to tissue regeneration [27,42,43].

Astragalus polysaccharide (APS), derived from the traditional Chinese medicinal plant Astragalus membranaceus, has gained attention for its potent immunomodulatory properties. APS has been extensively studied for its ability to modulate immune responses, particularly in inflammatory conditions and tissue repair. APS exerts its effects by promoting M2 macrophage polarization, enhancing anti-inflammatory signaling, and suppressing the release of pro-inflammatory cytokines like TNF-α and IL-6. In the context of bone regeneration, APS not only helps to dampen excessive inflammation but also promotes the secretion of cytokines such as IL-10, which is crucial for the resolution of inflammation and initiation of tissue repair [44,45].

In this study, the superior performance of the APS/PSBPK scaffold in promoting M2 polarization and reducing the secretion of pro-inflammatory cytokines can be attributed to the inclusion of APS. The APS/PSBPK scaffold, enriched with both bioactive ions and APS, represents a promising strategy for bone regeneration by integrating the immunomodulatory effects of Sr^2+^ and APS. This study highlights the ability of scaffold to effectively reduce pro-inflammatory responses while promoting an anti-inflammatory, pro-regenerative environment. The combination of bioactive ions (Sr^2+^) with APS enhances the ability of scaffold to promote M2 macrophage polarization and downregulate the expression of inflammatory markers, thus accelerating bone healing.

### 3.5. Effects of the Scaffolds on the Angiogenic Activity of HUVECs

**Figure 7:**
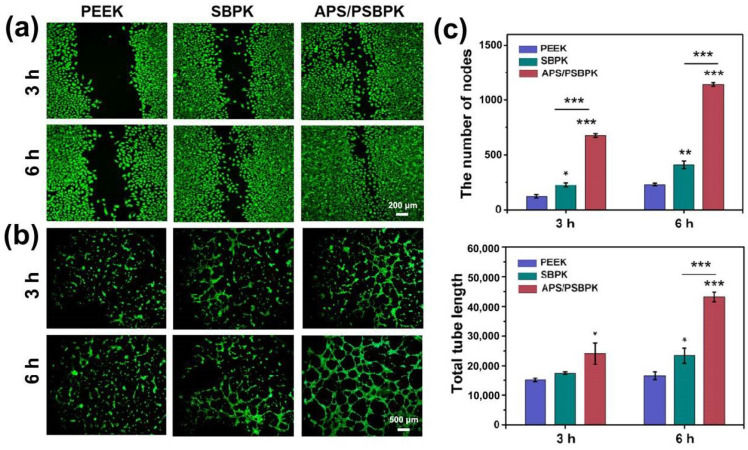
Proangiogenic activity of the different scaffolds in vitro. Representative images of scratch wound closure (**a**) and in vitro tube formation assay (**b**) of HUVECs cocultured with different scaffolds after 3 and 6 h. Quantitative analysis (**c**) of in vitro tube formation assay. (* *p* < 0.05, ** *p* < 0.01, *** *p* < 0.001).

Bone tissue repair requires effective vascularization to ensure sufficient nutrient and oxygen supply, and biomaterials can enhance angiogenesis and cell migration are valuable for promoting bone healing [46]. In this study, the angiogenic effects of PEEK, SBPK, and APS/PSBPK scaffolds were analyzed through HUVEC migration and tube formation assays to assess their suitability for bone tissue engineering (Figure 7). SBPK scaffolds, enriched with bioactive ions, demonstrated an improvement in HUVEC migration (Figure 7a) and angiogenic activity (Figure 7b) compared to PEEK, indicating that ion release from SBPK surfaces can stimulate endothelial cell motility and initiate early vascular network formation. The enhanced migration in the SBPK group highlights the role of ions in creating a more favorable microenvironment for endothelial cells, contributing to early-stage vascularization. APS/PSBPK scaffolds showed a marked enhancement in both HUVEC migration and tube formation relative to both PEEK and SBPK. APS/PSBPK scaffold promoted the formation of denser and more interconnected tubular structures, with significantly increased numbers of nodes and total tube length, suggesting that the scaffold has a pronounced effect on supporting endothelial cell behavior crucial for vascularization. The APS released from APS/PSBPK scaffold further enhances the bioactivity of SBPK, leading to accelerated cell migration and formation of complex capillary-like structures, essential for the vascular integration of bone grafts. Quantitatively (Figure 7c), APS/PSBPK scaffolds exhibited the highest values in node count and tube length compared to PEEK and SBPK scaffolds (*p* < 0.001), underscoring their potential as a proangiogenic material. The combined effects of ion release and surface bioactivity make APS/PSBPK a highly promising scaffold for applications requiring rapid and effective vascularization, crucial for bone defect repair.

### 3.6. In Vivo Experiment

**Figure 8:**
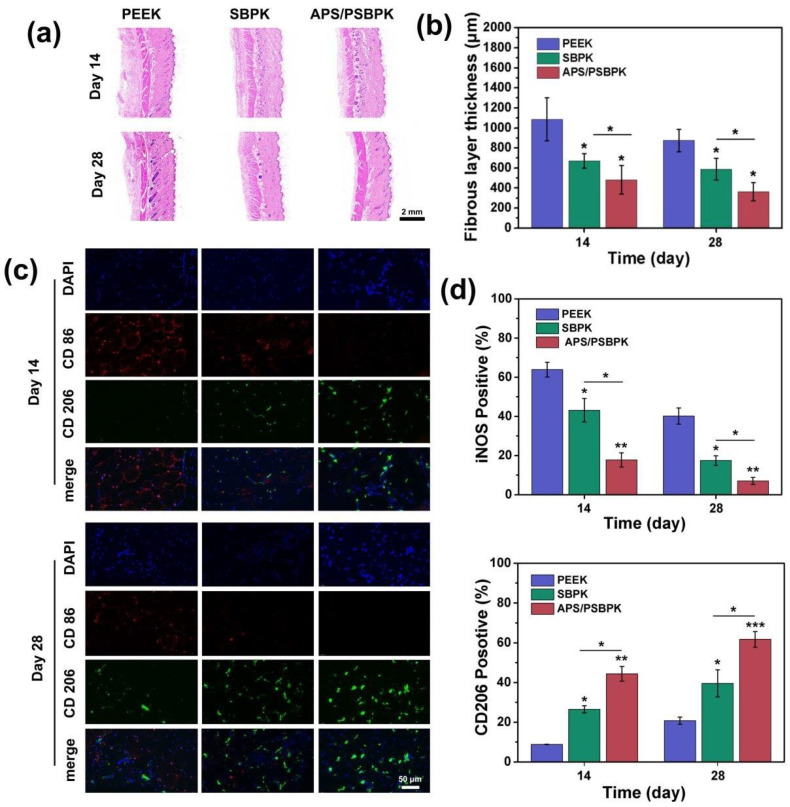
(**a**) Fibrous layer thickness around PEEK, SBPK, and APS/PSBPK scaffolds at 14 and 28 days; (**b**) quantification of fibrous layer thickness across different time points; (**c**) immunofluorescence staining of macrophage markers CD86 and CD206 in scaffold-surrounding tissues at 14 and 28 days; (**d**) percentage of iNOS and CD206 positive macrophages over time. (* *p* < 0.05, ** *p* < 0.01, *** *p* < 0.001).

To evaluate the immune response to the scaffolds in vivo, H&E staining and immunofluorescence analysis were conducted on the subcutaneous tissues surrounding the implants at 14 and 28 days. The fibrous layer (Figure 8a,b) surrounding the scaffolds shows a significant decrease in thickness as time progresses. Compared to the PEEK group, the SBPK and APS/PSBPK scaffolds exhibited substantially thinner fibrous layers, with APS/PSBPK scaffolds showing the most pronounced reduction at both 14 and 28 days (*p* < 0.05). A thinner fibrous layer is indicative of a more favorable host tissue response, suggesting that APS/PSBPK scaffolds are less prone to foreign body reactions, which is essential for improved scaffold integration and function in tissue repair.

Immunofluorescence staining was performed to evaluate the macrophage response around the scaffolds, specifically focusing on CD86 (a marker for pro-inflammatory M1 macrophages) and CD206 (a marker for anti-inflammatory M2 macrophages) expression (Figure 8c). CD86-positive (M1) macrophages decreased significantly around the SBPK and APS/PSBPK scaffolds by day 28, while the presence of CD206-positive (M2) macrophages increased substantially. Quantification of iNOS-positive cells (Figure 8d) further supports this finding, as the percentage of these pro-inflammatory cells decreased markedly in the APS/PSBPK group. Meanwhile, CD206-positive macrophages, which are associated with tissue repair and regeneration, increased significantly, with the highest percentage observed in the APS/PSBPK group at both time points.

These findings suggest that the APS/PSBPK scaffolds not only reduce fibrous encapsulation but also modulate the immune response by promoting the polarization of macrophages from the M1 to the M2 phenotype. This ability to shift the immune environment towards a reparative state highlights the potential of APS/PSBPK scaffolds for enhanced tissue regeneration and reduced inflammatory reactions, making them promising candidates for applications in bone tissue repair and other regenerative therapies.

In this study, neovasculature in the subcutaneous tissues at weeks 6 and 12 was assessed by immunohistochemical staining with an endothelial cell marker of CD31 (Figure S2a). Compared with PEEK or SBPK groups, increased vascularization was observed in the neobone induced by APS/PSBPK scaffolds, as indicated by a higher expression of CD31. Only few microvessels were observed in the tissues induced by PEEK or SBPK scaffolds. The release of APS from the scaffold plays a vital role in stimulating endothelial cells, facilitating the formation of well-organized, interconnected capillary-like structures. CD31 staining-based quantification revealed significantly higher microvessel density in the APS/PSBPK scaffold compared to PEEK and SBPK groups (*p* < 0.001) at both early and later time points (Figure S2b). Angiogenesis is essential in bone healing, as newly formed blood vessels provide a pathway for nutrients, oxygen, and growth factors necessary for cellular activities and tissue regeneration [47].

Moreover, the superior performance of the APS/PSBPK scaffold compared to standard PEEK or SBPK scaffolds underscores the importance of drug and/or bioactive ion release in designing effective bone repair materials. Their ability to enhance vascularization and regulate immune response suggests that APS/PSBPK scaffolds may significantly reduce healing time and improve outcomes in bone defect repair, making them an excellent candidate for further development in clinical applications.

**Figure 9:**
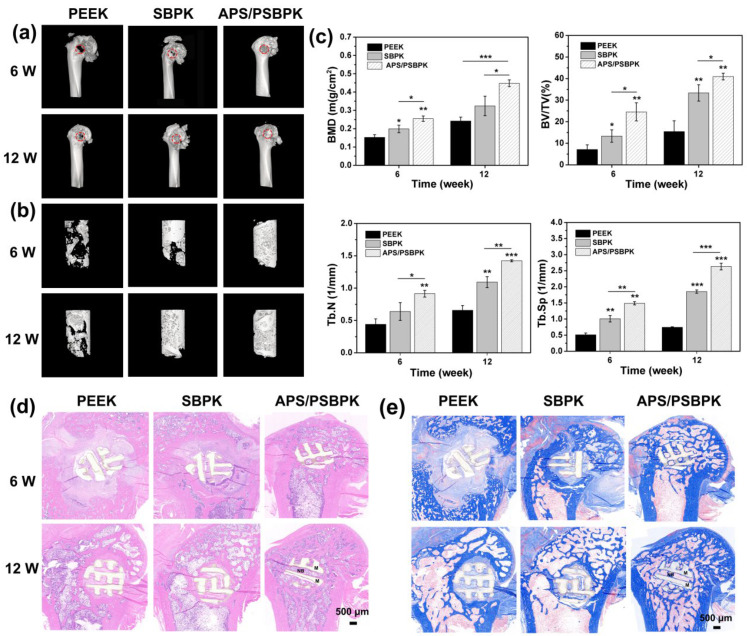
The μ-CT images of bone defects (**a**) and new bone regeneration (**b**) after scaffolds implantation 6 and 12 w in vivo. Quantitative analysis (**c**) of BV/TV, BMD, Tb.Th and Tb.N (**b**) of new bone in all groups. The H&E staining (**d**) and Masson’s staining (**e**) of bone defects after scaffolds implantation for 6 and 12 weeks. (red circles: bone defects, M: materials, NB: new bone) (* *p* < 0.05, ** *p* < 0.01, *** *p* < 0.001).

To evaluate the effect of different scaffold materials on bone repair, μ-CT images (Figure 9a,b) were captured from the bone defect sites after 6 and 12 weeks of implantation. Additionally, quantitative analyses of bone mineral density (BMD), bone volume fraction (BV/TV), trabecular number (Tb.N), and trabecular separation (Tb.Sp) were performed to assess new bone formation across the PEEK, SBPK, and APS/PSBPK scaffolds (Figure 9c). At 6 weeks, the PEEK group showed minimal new bone formation, with low BV/TV and BMD values, reflecting limited early-stage bone regeneration. In contrast, both the SBPK and APS/PSBPK groups exhibited more moderate bone growth, but the APS/PSBPK scaffold demonstrated significantly superior bone formation, as indicated by higher BV/TV and BMD values (*p* < 0.01). Furthermore, the APS/PSBPK group had a notably higher Tb.N and lower Tb.Sp compared to the PEEK group (*p* < 0.01), suggesting a more robust trabecular structure and better bone connectivity at this early time point. By 12 weeks, the differences between the groups became more pronounced. The APS/PSBPK scaffold continued to outperform both the PEEK and SBPK groups, with significantly higher BV/TV and BMD levels, reflecting superior long-term bone regeneration. While the SBPK group showed improvement from 6 weeks, its bone formation was still less pronounced than that of the APS/PSBPK group, with lower BV/TV, BMD, and Tb.N values, as well as higher Tb.Sp. This indicates that the APS/PSBPK scaffold facilitated a more favorable microarchitecture for bone healing, with denser trabecular connectivity and a more stable bone structure compared to both the PEEK and SBPK groups.

Hematoxylin and eosin (H&E, Figure 9d) and Masson’s trichrome staining (Figure 9e) were conducted on bone defect sites after 6 and 12 weeks of implantation to assess the effectiveness of PEEK, SBPK, and APS/PSBPK scaffolds in promoting bone repair. At 6 weeks, minimal new bone tissue formation was observed in the PEEK group, with limited cellular activity around the defect area. In the SBPK scaffold, there was moderate formation of new bone tissue, indicating some early osteogenic response. In contrast, the APS/PSBPK scaffold exhibited a noticeable increase in new bone tissue, suggesting an accelerated healing process and enhanced osteoinductive potential at this early stage. By 12 weeks, a more pronounced difference in new bone formation was evident. The PEEK scaffold continued to show limited bone tissue growth, suggesting insufficient osteoconductivity. The SBPK scaffold demonstrated increased new bone tissue compared to the APS/PSBPK scaffold. In the APS/PSBPK group, a substantial amount of new bone tissue had developed around the scaffold structure, with clear integration into surrounding bone tissue. This scaffold displayed mature bone matrix formation, indicating strong osteogenic capability and effective support for bone defect healing. Thus, the APS/PSBPK scaffold is significantly more effective in promoting bone regeneration compared to both PEEK and SBPK scaffolds.

Bone regeneration is a complex and systemic process that cannot be effectively replicated by a single factor [48]. Compared with PEEK and SBPK scaffolds, APS/PSBPK scaffolds exhibited multiple beneficial functions, including the promotion of macrophage polarization towards the M2 phenotype and the enhancement of both angiogenesis and osteogenesis, contributing to biomimetic bone repair. These effects were primarily achieved through the controlled release of APS and bioactive ions. Initially, APS was rapidly released from APS/PSBPK scaffolds to modulate immune responses and stimulate angiogenesis, which subsequently facilitated bone formation. Meanwhile, the gradual release of bioactive ions from the scaffolds further promoted osteogenic differentiation. Angiogenesis plays a crucial role in the bone healing process, as newly formed blood vessels supply oxygen, nutrients, and essential growth factors required for osteogenesis. These vessels may also transport immune cells, stem cells, and progenitor cells to the injury site, supporting tissue regeneration. Additionally, the enhanced polarization of macrophages towards the M2 phenotype helps mitigate inflammation and fosters bone tissue regeneration. The combined effects of immune regulation and vascularization further amplify the osteogenic potential induced by bioactive ions, thereby enhancing bone repair.

Compared to existing studies on PEEK composites [17,18,20,21,49], this study offers a more comprehensive evaluation by dynamically simulating the in vivo environment. This dynamic assessment provides deeper insights into the interaction of scaffold with biological systems over time, particularly in terms of osteogenesis, immune modulation, and angiogenesis. In conclusion, APS/PSBPK scaffolds establish a dynamic, biomimetic microenvironment that supports bone regeneration through multiple synergistic mechanisms, making them highly promising for future clinical applications.

However, this study has certain limitations. The animal model used in this study effectively evaluates early and mid-stage bone repair, but differences remain between its bone regeneration environment and that of the human body, particularly in terms of adaptation under load-bearing conditions, which requires further validation. Additionally, the experimental period is relatively short, limiting the assessment of long-term scaffold integration and stability after implantation. Moreover, future research would further explore the specific mechanisms of APS/PSBPK scaffolds in osteogenesis, immune modulation, and angiogenesis to deepen the understanding of their biological properties. This will help optimize scaffold design and enhance its clinical potential for bone repair applications.

## 4. Conclusions

In this study, an innovative APS/PSBPK composite scaffold with a well-defined three-dimensional porous structure (~5 μm) and a square channel architecture (approximately 400 × 400 μm) was successfully developed. The scaffold demonstrated enhanced hydrophilicity and superior mineralization potential. The APS/PSBPK scaffold exhibited excellent immunomodulatory capabilities by shifting macrophage polarization from the pro-inflammatory M1 phenotype to the anti-inflammatory M2 phenotype, thereby reducing inflammatory cytokine production and promoting the release of anti-inflammatory mediators. The scaffold also significantly supported vascularization, as evidenced by improved HUVEC migration and tube formation. Additionally, it facilitated osteogenic differentiation by promoting rBMSC adhesion, proliferation, and the upregulation of key osteogenic markers such as ALP activity and calcium nodule deposition. In vivo subcutaneous implantation studies confirmed the scaffold’s ability to regulate the immune microenvironment and promote vascularization. In a rat femoral defect model, APS/PSBPK scaffolds demonstrated superior bone regeneration. These findings suggest that the APS/PSBPK composite scaffold holds significant potential as a bioactive material for bone repair applications due to its ability to modulate the bone immune microenvironment and promote angiogenesis and osteogenesis. Future studies would further explore its long-term stability and mechanical performance under load-bearing conditions, as well as its for clinical applications. Additionally, a deeper exploration of the underlying mechanisms governing its osteogenesis, immune modulation, and angiogenesis is needed to optimize its biological performance.

## Data Availability

All supporting data are contained within the manuscript and Supplementary Materials.

## Supplementary Materials

The following supporting information can be downloaded at: https://www.mdpi.com/article/10.3390/polym17091206/s1, Figure S1: SEM images (a) and EDS spectra (b) of PEEK, SBPK, APS/PSBPK scaffolds after immersion in SBF for 14 days; Figure S2: Immunohistochemical staining of CD31 protein expression (a) and quantification of CD31+ micro-vessels (b) at 14 and 28 days. (** *p* < 0.01, *** *p* < 0.001); Table S1: Mechanical properties of the scaffolds.
