# Supplementary material for: A Multifunctional PEEK Composite Scaffold with Immunomodulatory, Angiogenic, and Osteogenic Properties for Enhanced Bone Regeneration
Source: Polymers (Basel). 2025 Apr 28;17(9):1206. doi: 10.3390/polym17091206 (PMC12073393; doi:10.3390/polym17091206)
Supplement: Supplementary file 1 [file polymers-17-01206-s001.zip › polymers-3508132-SI.pdf]

Supplementary Material

A Multifunctional PEEK Composite Scaffold with Immunomodulatory, Angiogenic, and Osteogenic Properties for Enhanced Bone Regeneration

Mengen Zhao[1,2], Han Yang[1], Qianwen Yang[2], Chao Zhang[2], Zhaoying Wu[2], Lijun Wang[1], Wei Zhang[1], Bing Wang[1*] , Wenliang Liu[1*] and Jie Liu[2 *]

[1]Shenzhen Institute for Drug Control, Shenzhen Testing Center of Medical Devices, Shenzhen, Guangdong, 518057, China

[2] School of Biomedical Engineering, Shenzhen Campus, Sun Yat-Sen University, Shenzhen, Guangdong, 518107, China

Correspondence should be addressed to:

E-mail: zhaomen3@mail.sysu.edu.cn

1. Materials and Methods

1.1 Materials

PEEK powder (99%, Junhua PEEK, China), tetraethyl orthosilicate (TEOS, Tianjin Zhiyuan Reagent, China) cetyltrimethylammoniumchloride (CTAC, Tianjin Zhiyuan Reagent, China), ammonium hydroxide ($NH_3 \cdot H_2O$, 28%, Tianjin Zhiyuan Reagent, China), calcium nitrate tetrahydrate ($Ca(NO_3)_2 \cdot 4H_2O$, 99%, Aladdin, China), and strontium tetrahydrate ($Sr(NO_3)_2$, 98%, Aladdin, China), stragalus polysaccharide (APS, Sigma, USA), Cell Counting Kit-8 (CCK-8, Beyotime, China), Triton X-100 solution (Amresco, USA), paraformaldehyde solution (4%, Seville Creature, China), alizarin red dye solution (2%, ScienCell, USA), cetylpyridinium chloride (Sigma, USA), bovine serum albumin (BSA, Beyotime, China), BCIP/NBT ALP color development kit (ALP, Beyotime, China), RITC-conjugated Phalloidin (Abcam, UK) and 4′,6 diamidino-2-phenonylindole (DAPI, Solarbio, China), PE anti-mouse CD86 antibody (Biolegend, USA), FITC anti-mouse CD206 (MMR) antibody (Biolegend, USA), TNF-α ELISA kit (Jianglaibio, China), IL-10 ELISA kit (Jianglaibio, China), live/dead staining kit (EFLsensei, China), Matrigel (BD Biosciences, USA), hematoxylin/eosin (H&E) staining kit (Solarbio, China), and Masson's trichrome staining solution (Solarbio, China) were used as received. All other solvents are of analytical grade and used without purification.

1.2. Cells and animals

Dulbecco's modified Eagle Medium/Nutrient mixture F-12 (DMEM/F12, Gibco, USA) supplemented with 10% of fetal bovine serum (FBS, Hyclone, USA), 1% of

penicillin and streptomycin (Pen/Strep, Gibco, USA) was used as the growth medium for rat bone marrow stromal cells (rBMSCs); DMEM/F12 supplemented with 10% of FBS, 1% of Pen/Strep, 10 mM of β-glycerolphosphate (Sigma, USA), 100 nM of dexamethasone (Sigma, USA), and 0.05 mM of ascorbic acid (Sigma, USA) was used as the osteogenic medium for rBMSCs; DMEM (Gibco, USA) supplemented with 10% of FBS and 1% of Pen/Strep was used as growth medium for murine preosteoclast cells (RAW 264.7, Procell, China) and Human Umbilical Vein Endothelial Cells (HUVECs) .

The rat bone mesenchymal stem cells (rBMSCs) were isolated from the femoral bone marrow of Sprague Dawley (SD) rat (80 g) (Laboratory Animal Center of Shenzhen Institute for Drug Control, Shenzhen Testing Center of Medical Devices, Shenzhen, China) and cultured in growth medium at 37 °C in a humidified CO_2 incubator. All the cells were routinely sub-cultured upon reaching approximately 80% confluence.

SD rats (8 weeks, 200−250 g) from the Experimental Animal Centre of Shenzhen Testing Center of Medical Devices (Shenzhen, China) were used in this study. All animal experiment protocols were approved by the Institutional Animal Care and Use Committee of Shenzhen Institute for Drug Control (Shenzhen Testing Center of Medical Devices).

2 Methods

2.1 Water contact angle

The water contact angles of PEEK, SBPK and APS/PSBPK scaffolds were

determined using a contact angle measuring instrument via the suspension drop method. A 10 μL droplet of water was placed on each scaffold, and the system controlled the droplet volume and drop rate. The dynamic tracking mode was used to continuously monitor the droplet, and images were captured for analysis. The contact angles were then calculated using DSA1A software based on the processed images.

2.2 Water contact angle

The mechanical properties of the scaffolds (Φ6×6 mm) were evaluated using a universal testing machine (INSTRON 5982, USA) at a fixed strain rate of 0.5 mm/min to determine their compressive strength and modulus of the scaffolds.

2.3 In vitro mineralization and ion release from scaffolds

The PEEK, SBPK, and APS/PSBPK scaffolds were immersed in 2.0 mL of simulated body fluid (SBF) solution and placed in a shaking incubator at 37 °C. After 14 days of immersion, the samples were collected, rinsed with deionized water, and dried at 60°C for 24 hours. The surface morphology and composition of the samples were characterized using field-emission scanning electron microscopy (FESEM). Additionally, the concentrations of Ca^{2+}, Sr^{2+} and SiO_3^{2-} ions in the SBF solution at different time points were measured using inductively coupled plasma optical emission spectroscopy (ICP-OES).

2.4 APS release from scaffolds

The composite scaffolds were immersed in 2.0 mL of PBS solution and placed in a shaking incubator at 37.0°C. At predetermined time points, 200 μL of the release medium was collected and replaced with fresh PBS solution. The amount of APS

released from APS/PSBPK scaffold into the solution was measured using a total polysaccharide content assay kit (phenol-sulfuric acid method).

2.5 The roughness of the scaffolds

A 3D laser confocal microscope was used to capture the laser intensity of the scaffold surface at two different magnifications (200x and 1000x), under a 658 nm laser. The collected laser intensity data was processed using VK-H1XAC 3D reconstruction software to generate a three-dimensional image and height distribution of the scaffold surface. The surface roughness of the scaffolds was also analyzed based on the data.

2.6 Immunofluorescence staining

The immunofluorescence staining process for 2.0×10^5 RAW 264.7 cells cultured on the scaffold surface for 3 days was conducted as follows. The cells were fixed with 4% paraformaldehyde for 30 minutes at room temperature, followed by PBS wash for three times. Then, 200 μL of PE-anti-CD86 antibody (diluted 1:400 in PBS) was applied, and the cells were incubated in the dark at 4°C for 20 minutes. After a subsequent PBS wash, 200 μL of FITC-anti-CD206 antibody (diluted 1:400 in 0.1% saponin solution) was added for another 20 minutes. Then, the cells were stained with 200 μL of DAPI solution for 10 minutes at room temperature in the dark. The samples were then imaged using a confocal laser scanning microscope.

2.7 ELISA analysis

RAW 264.7 cells (2.0×10^5) were seeded onto the scaffold surface and cultured for 1, 3, and 5 days. The cell culture media from different scaffolds were collected and centrifuged at 1000 rpm for 5 minutes to obtain the supernatant. The levels of tumor

necrosis factor-alpha (TNF-α) and interleukin 10 (IL-10) in the supernatant were measured using a mouse TNF-α ELISA kit and an IL-10 ELISA kit.

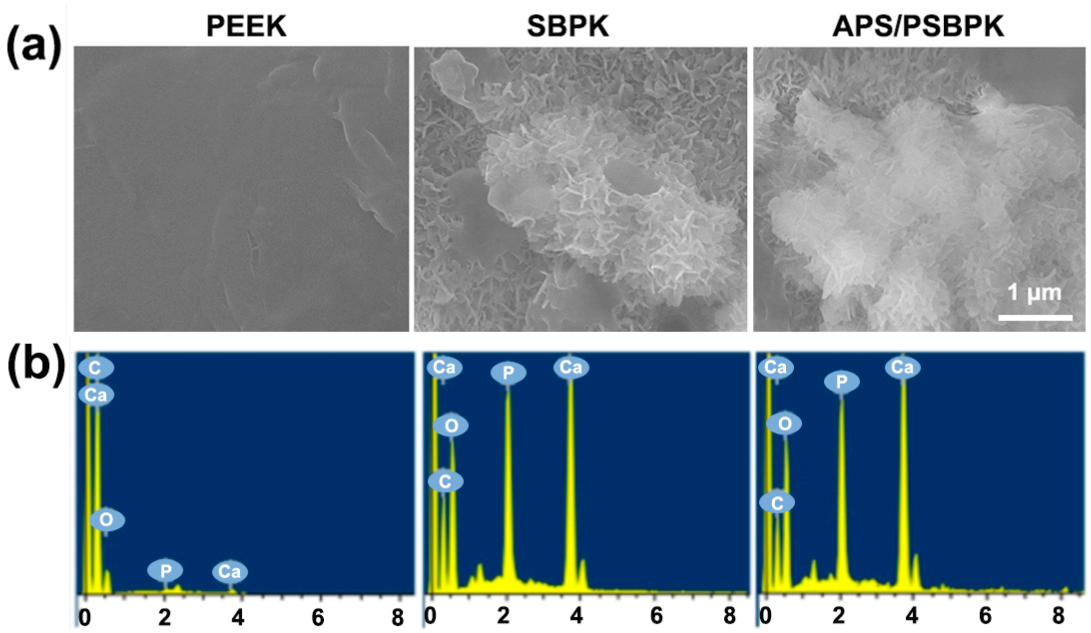

Figure S1 SEM images (a) and EDS spectra (b) of PEEK, SBPK, APS/PSBPK scaffolds after immersion in SBF for 14 days.

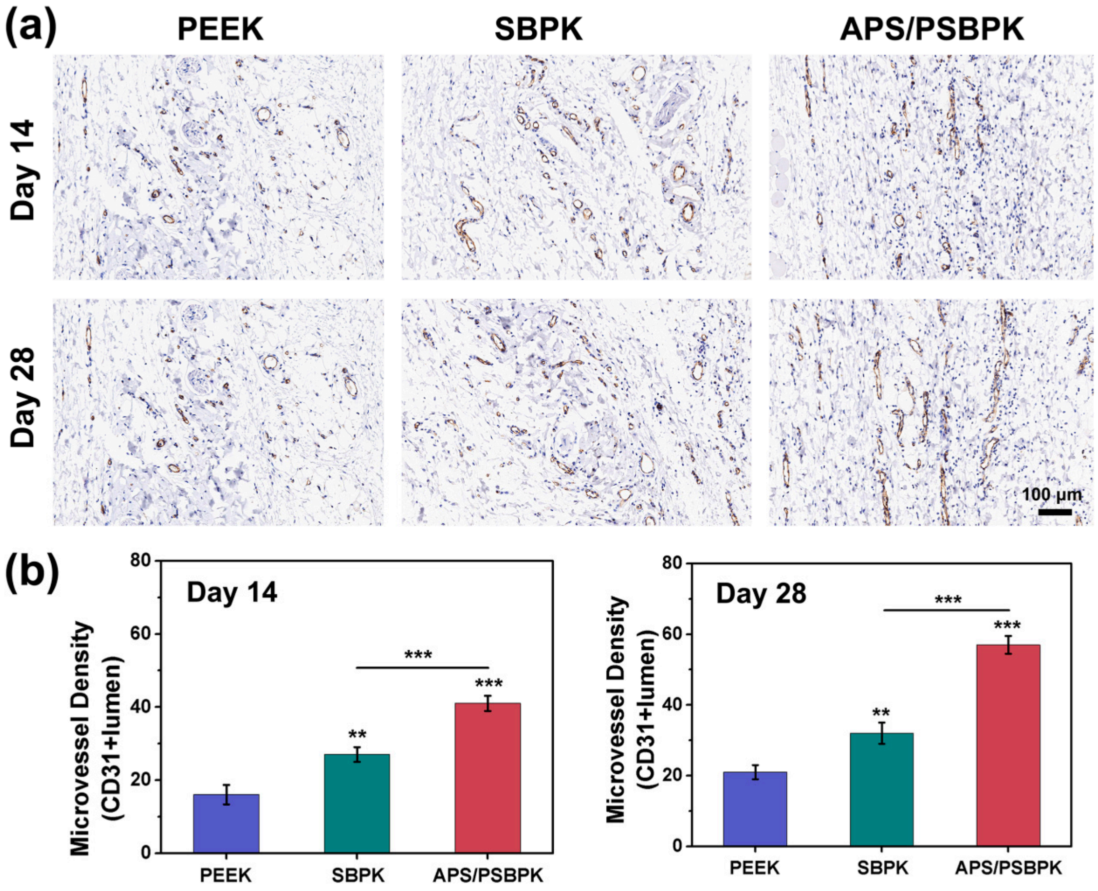

Figure S2 Immunohistochemical staining of CD31 protein expression **(a)** and quantification of CD31+ micro-vessels **(b)** at 14 and 28 days. (**p < 0.01, ***p < 0.001)

Table S1 Mechanical properties of the scaffolds.

Scaffolds	Compressive strength (MPa)	Modulus of elasticity (MPa)
PEEK	35.24±0.65	585.33±8.14
SBPK	32.10±1.20	527.67±7.02
APS/PSBPK	32.99±1.79	531.00±3.61
